# Vaginal Drain to Prevent Ascending Infection in Preterm Premature Rupture of Membranes: A Novel Method

**DOI:** 10.7759/cureus.42204

**Published:** 2023-07-20

**Authors:** Hemmanoor Samartharam, Sai Samyuktha Ila, Nagashree Vasudeva

**Affiliations:** 1 Obstetrics and Gynecology, Sandhyaram Maternity Hospital, Palakkad, IND; 2 Obstetrics and Gynecology, Divya Hospital, Thirpur, IND

**Keywords:** a novel method, prevent chorioamnionitis, ascending infection, preterm premature rupture of membranes, vaginal drain

## Abstract

Background: Not to delay delivery for more than 24 hours after rupture of the membrane is the thumb rule in obstetrics. Ascending infection from the vagina results in dangerous chorioamnionitis, which threatens the lives of the baby and mother. We developed a novel method to prevent ascending infection by using a vaginal drain, which helps to continue pregnancies if the leak stops and buys some time for antenatal steroids to act if the leak doesn’t stop.

Method: In this study, 20 uncomplicated singleton pregnant women with spontaneous preterm premature rupture of membranes at gestational ages <35 weeks were recruited. Under the speculum, vaginal epithelial debris and secretions were cleared by a saline wash. The tip of the Nelaton Catheter was kept in the posterior fornix and then strapped to the thigh. The outer end was connected to the collection bag system and allowed to hang down to the ground. The foot end of the cot was raised. Leaked amniotic fluid and amniotic fluid index (AFI) were measured daily. A daily AFOD estimation was done for leaked AF. Uterine activity was controlled with low-dose Isoxsuprine Hydrochloride rapid infusion tocolysis. Antenatal steroids (ANS) were given. The spread of infection was monitored by maternal pulse rate, fetal heart rate, TC, and CRP. Pregnancies were terminated when leaks didn’t stop, AFI didn’t raise, or mature AFOD was observed. The number of women in whom leaks stopped, the number of days pregnancies continued, neonatal respiratory distress (NRD), birth weights, and perinatal deaths were recorded.

Results: Leaks stopped in eight (40%) women. Pregnancy continuation ranged from 7 to 74 days. In 12 women, the leak did not stop, but we could buy 2-5 days’ time for ANS to act. All parameters of the infection were within normal limits. Thirteen babies developed mild NRD, and we lost one baby.

Conclusion: By using a vaginal drain for P(P)ROM, ascending infection can be prevented, leaks can be stopped in 40% of women, and can buy time for ANS to act.

## Introduction

Preterm premature rupture of membranes (PPROM) complicates 3-10 percent of pregnancies and leads to one-third of preterm deliveries [1-4]. Prematurity is the leading cause of neonatal mortality and morbidity and also predisposes to long-term morbidities like diabetes mellitus, hypertension, obesity, and stroke [5]. As many as 15 million preterm births occurred worldwide in the year 2010, as per the Lancet report [6]. The global action report by WHO, “Born too soon,” says the incidence of preterm birth is progressively increasing worldwide [7].

The general guideline in obstetrics is to avoid delaying delivery for more than 24 hours following the rupture of the membrane. Ascending infection from the vagina results in dangerous chorioamnionitis, which threatens the lives of the baby and mother. The main objectives in the management of PPROM include stopping uterine activity, sealing the leaking membrane to prevent ascending infection, improving the volume of liquor, and hastening the fetal functional maturity with antenatal steroids (ANS). In case the leaking could not be stopped, at least we could buy some time for the antenatal steroids to act to enhance overall fetal functional maturity and prevent respiratory morbidity. Earlier small studies reported the technique of ‘Amniopatch’ in which amnioinfusion of autologous platelet concentrate and cryoprecipitate seals the leaking membrane in cases of spontaneous PPROM. The success rates were only 11.8% to 14.3% [8,9].

In this study, we used a novel method of using a vaginal drain to prevent ascending infections. This approach aids in either prolonging pregnancies if the leakage can be controlled or providing a window of time for antenatal steroids to take effect if the leakage cannot be stopped.

## Materials and methods

Hypothesis and proposed mechanism for how it works

When the membrane ruptures, leaked amniotic fluid accumulates in the fornices around the cervix. This leaked fluid can become infected with vaginal pathogens, turning it into septic material. The loss of amniotic fluid from the uterine cavity triggers uterine contractions. These contractions, followed by relaxation, create changes in pressure inside the uterus, leading to the back-and-forth movement of the septic liquor between the uterine cavity and the fornices. This movement of infected fluid increases the risk of ascending infection, chorioamnionitis, and its complications.

The use of a vaginal drain in cases of P(P)ROM helps address this issue. The drain system facilitates the suctioning of the leaked and infected fluid, utilizing capillary action and gravity acting on the fluid column within the collection tube. This process aims to prevent the occurrence of ascending infections and chorioamnionitis by removing the septic fluid.

Methods

**Figure 1:**
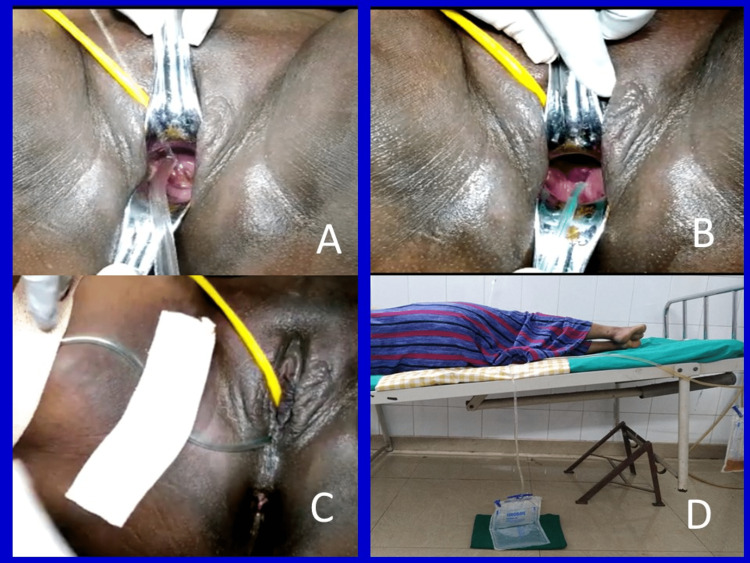
Cleaning the vagina under a saline jet and suction (A); Tip of the Nelaton urethral catheter kept in the posterior fornix (B); Nelaton Catheter strapped to the thigh and connected to the drainage system. (C); Drainage system and the collection bag touching the ground (D)

In this preliminary observational study, 20 uncomplicated singleton pregnant women with spontaneous preterm premature rupture of membranes (PPROM) at gestational ages <35 weeks were recruited. Women with membrane ruptures lasting more than six hours were excluded from the study. Informed and written consent was obtained from all subjects who participated in this study. This study was confined to the standards of the Declaration of Helsinki. Under aseptic precautions, leaking was confirmed by speculum examination. The amniotic fluid index (AFI) was recorded for all women. After application of a vaginal speculum using a saline jet and suction tip, vaginal epithelial debris and secretions were cleared. The tip of a Nelaton urethral catheter was kept at the posterior fornix, and the speculum was removed gently. A Nelaton catheter was strapped to one of the thighs. The outer end of the urethral catheter was connected to the collection bag system, and the bag was allowed to hang down to the ground (Figure 1 and Video 1). Leaked amniotic fluid that was collected in the bag was measured at 24-hour intervals. The collection bags were changed every day. Cleaning of the vagina was done on alternate days. Amniotic Fluid Optical Density (AFOD) estimations were done for fresh AF samples collected from the suction tube daily (Video 2). Foley’s catheterization of the bladder was done for continuous bladder drainage. The foot end of the cot was raised by 1.5 feet, and the women were made to lie down in the left lateral or supine position, whichever position was comfortable for them. A bed pan was given for passing stools to prevent ambulation.

**Video 1:** Cleaning the vagina using a saline jet and suction tip

**Video 2:** AF sample collection and AFOD estimation

Uterine activity was controlled by low-dose Isoxsuprine Hydrochloride rapid infusion tocolysis [10]. Intravenous antibiotics (Inj. Cefazolin, Inj. Gentamycin, and Inj. Metronidazole) were given in adequate doses to cover gram-positive, gram-negative, and anaerobic organisms. The spread of sepsis was monitored by recording maternal temperature, pulse rate, and fetal heart rate every eight hours. Total leucocyte counts and C-reactive protein (CRP) were done daily. Antenatal steroids were given. Pregnancies were terminated when the leak did not stop, the AFI did not rise within 72 hours, or the parameters of infection were raised, or when the AFOD value reached more than 0.60 [11]. The collected amniotic fluid volume in the bag was graded as Grades 1, 2, 3, and 4. If the collected liquor volume was <10 ml: Grade 1; between 11 ml and 50 ml: Grade 2; between 51 ml and 100 ml: Grade 3; and if > 100 ml: Grade 4. The AFI was also graded as Grades 1, 2, 3, and 4. If AFI < 8cm: Grade 1, between 8.01 cm and 12.00 cm: Grade 2, between 12.01 cm and 16.00 cm: Grade 3, and if > 16 cm: Grade 4. The study also gathered information about the trend of changes in grades of AFI and the trend of changes in the grades of collected liquor in the bag in selected five women of the study, with cessation of the leak and without cessation of the leak. 

Women were ambulated gradually after complete cessation of leakage and satisfactory control of uterine activity. Women were discharged when they were clinically stable without leakage. Inj. 17 α Hydroxyprogesterone Caproate 500mg IM weekly was given. If the uterus was irritable at the time of discharge, 10mg of Tab Isoxsuprine Hydrochloride was given three times daily for a week. Recurrent rupture of membranes was treated along the same lines. Women were advised to take more than 3 Lt of oral fluids/day. Intravenous L-arginine infusion, 300 ml, was given on alternate days to improve liquor [12].

The number of women for whom leaks could be stopped and the number of women for whom leaks could not be stopped were recorded. The number of days that pregnancies could continue in women with and without cessation of leakage was recorded and shown in Table 1. Recorded maternal temperature, pulse rate, fetal heart rate, Total leucocyte counts, and C-reactive protein (CRP) are shown in Table 2. Birth weights, neonatal respiratory distress, the number of NICU admission days required, and the perinatal outcome for each woman were recorded and shown in Table 3.

Cleaning the vagina using a saline jet and suction tip

Cleaning the vagina was done to reduce the bacterial load and minimize ascending infections. A saline bottle was perforated at the top end with a large-bore needle. A saline jet was produced by squeezing the bottle. A suction machine, suction tube, and suction tip were organized. Under strict aseptic precautions and a good source of light, a vaginal speculum was applied, and the cervix and vagina were visualized. By using the saline jet and suction tip simultaneously, epithelial debris and mucus secretions were removed from the vagina (Figure 1 and Video 1). This procedure was repeated on alternate days. 

Low-dose Isoxsuprine hydrochloride rapid infusion tocolysis: In this technique, 10 mg (1 amp) of Isoxsuprine Hydrochloride in 500 ml of dextrose saline or Ringers lactate is run at a rate of 160-180 drops/min, delivering 200 micgr of beta-mimetic drug/min. Initially, we ran 2-3 pints of this fluid at a rate of 160-180 drops/min (10ml/min) to achieve quick tocolysis. Later, the infusion was given by an infusion pump, and the rate was adjusted to 300ml/hour, 200m/hour, or 100ml/hour, or the infusion was stopped based on the tocolysis response [10]. After complete cessation of uterine activity (usually 12 to 24 hours), two pints of the same infusion fluid were given at a rate of 160-180 drops/min, twice daily for 2 to 3 days. 

Method of measuring AFOD: The colorimeter was set at a 650 nm wavelength. The test tube containing distilled water (the control solution) was inserted into the cuvette holder of the machine, and the ‘0’ reading was adjusted. Then the control test tube was removed from the cuvette holder, and then the test tube containing a fresh, uncentrifuged AF sample was inserted. With the press of the enter button, the AFOD value can be read directly from the display screen of the machine [11]. AFOD estimation with a colorimeter can be seen in Video 2.

## Results

In eight women (40%), the amniotic fluid leak completely stopped, and the AFI started raising. These women could continue pregnancies for a variable period of 7 to 74 days (Table 1).

**Table 1:** Clinical details of 20 women with PPROM who were treated with a novel vaginal drain method to prevent ascending infection. We can see a progressive increase in AFI in the leak-sealed group and a progressive diminishing of AFI in the leak-not-sealed group. PROM: Preterm rupture of membranes; GA: Gestational age; AFI: Amniotic fluid index.

Sl. No.	GA at PROM Wks+d	Day wise, Liquor collected in drain bag ml						Day-wise AFI cm						Preg. Cont. days		
		Day 1	Day 2	Day 3	Day 4	Day 5	Day 6	Day 1	Day 2	Day 3	Day 4	Day 5	Day 6			
1	32+ 1	50	25	15	10	0	-	14.7	15.01	14.3	16.1	18.0	19.0	9		
2	21+ 6	50	25	15	10	-	-	14.7	15.01	14.3	16.4	-	-	74		
3	35+ 5	150	75	15	10	0	-	12.7	15.01	14.3	16.5	18.27	-	20		
4	34+ 2	130	80	25	10	-	-	13.65	16.01	14.3	16.5	-	-	11		
5	27+ 1	80	30	30	10	-	-	13.76	16.8	15.7	16.7	-	-	75		
6	34+ 3	120	80	35	10	-	-	11.36	16.21	14.3	16.6	-	-	7		
7	33+ 5	120	75	15	10	0	-	12.5	16.01	14.4	15.5	18.27	-	11		
8	32+ 1	65	30	15	10	0	-	14.7	15.01	14.1	16.3	16.1	-	9		
Women, in whom leaks did not stop																
9	34+ 1	80	70	15	-	-	-	4.3	10,4	5.67	-	-	-	3		
10	33+ 1	100	100	10	-	-	-	6.6	10.4	5.21	-	-	-	3		
11	35+ 0	75	30	20	-	-	-	16.2	11.4	4.89	-	-	-	4		
12	35+ 1	90	70	15	-	-	-	4.3	9.4	5.67	-	-	-	3		
13	28+ 4	75	100	10	-	-	-	16.6	10,4	4.22	-	-	-	4		
14	35+ 5	100	50	10	-	-		16.2	11.4	5.21	-	-	-	2		
15	33+ 4	120	70	25	15	10	0	12.7	15.01	14.3	16.5	12.27	5.27	5		
16	34+ 4	60	70	30	15	10	-	14.06	12.01	10.3	16.5	8.27	-	4		
17	31+ 2	120	70	25	15	10	0	13.8	15.03	14.3	15.5	12.27	5.26	5		
18	34+ 5	120	70	25	15	0	-	12.7	15.01	14.3	16.5	5.27	-	4		
19	35+ 3	80	60	10	-	-	-	4.5	9,4	5.67	-	-	-	4		
20	30+ 4	70	90	10	-	-	-	16.6	10,4	4.22	-	-	-	4		

Four of the babies of these women were fully functionally mature and did not develop NRD. Four babies developed mild NRD and responded well to oxygen supplementation with a mask. Birth weights ranged from 1.23 to 3.1 kgs (Table 3). One mother's membrane ruptured twice, once at 21 weeks + 6 days and again at 27 weeks + 4 days. At 28 weeks + 1 day, given limited resources, we referred the woman to the higher hospital. Given the adverse prognosis, labor was induced and delivered vaginally. The birth weight was 1.1 kg. She developed mild NRD, which responded well to oxygen supplementation with a mask. But the baby succumbed to death due to sepsis on the fifth day. 

**Figure 2:**
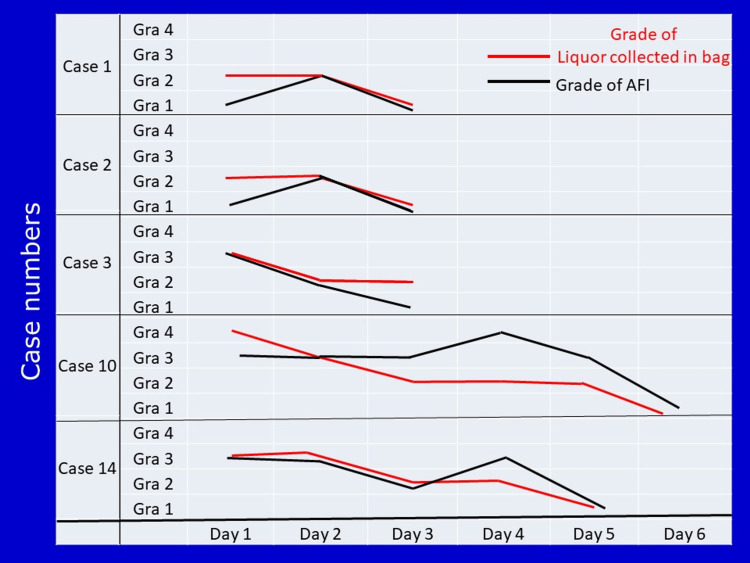
The trend of change in grade of collected liquor, and grade of AFI in selected five women, in whom the leak could not be stopped is shown.

In these eight women, a progressive increase in Grades of AFI and a progressive decrease in grades of collected liquor volume in the bag were observed (Table 1). The trend of change in AFI and the trend of change in the volume of liquor collected in the bag is shown in five selected cases in Figure 2.

We could continue pregnancies in all these women for a variable period of 7 to 74 days. In all eight mothers, all parameters of infection were within normal limits (Table 2). 

**Table 2:** Parameters of infection to assess the spread of infection in the study population. Parameters of the spread of infection in the study population are shown. PROM: Preterm rupture of membranes; GA: Gestational age; TLC: Total leucocyte count; CRP: C-reactive protein; FHR: Fetal heart rate.

Women, in whom leak sealed								
Sl. No.	GA at PROM W+D	GA at deliver W+D	Maternal temper: range	TLC: Range	CRP: Range	Mat pulse: Range	FHR: Range	Preg. Contin. Days
1	32+ 1	32+ 3	98.4- 99.9	7500- 15600	6.2- 10.7	87- 104	144- 156	9
2	21+ 6	28+ 1	98.5- 99.2	7800- 12,100	8.2- 11.1	76-97	140-160	74
3	35+ 5	38+ 4	98.6- 99.6	6,600- 14600	5.6- 10.2	86- 97	126- 170	20
4	34+ 2	35+ 6	98.4- 98.7	7,600- 13600	7.7- 9.9	72- 96	120- 159	11
5	27+ 1	37+ d	98.4- 100.6	7800-18000	6.8- 13.1	78-102	126-159	75
6	34+ 3	35+ 3	98.5- 99.8	8200- 13000	8.7- 12.1	87- 102	120-150	7
7	33+ 5	35+ 2	98.7- 99.0	9,500- 12700	8.1- 10.1	82- 102	135- 170	11
8	32+ 1	32+ 3	98.4- 98.9	7800- 15400	6.1-10.3	82- 103	144- 157	9
Women, in whom leaks could not be stopped								
1	34+ 1	34+ 4	98.4- 98.9	6800- 12,100	7.9-10.9	76-87	130-150	3
2	33+ 1	34+ 3	98.5- 99.2	12800- 21200	9.2- 16.7	61- 78	140-160	3
3	35+ 0	35+ 4	98.6- 100.2	10,600- 14600	9.1- 14.7	84- 98	138- 170	4
4	35+ 1	35+ 4	98.4- 99.3	9,600- 12600	8.2- 11.3	76- 102	136- 170	3
5	28+ 4	30+ 1	98.4- 100.2	10,800- 15600	6.1- 14.1	82- 100	141- 152	4
6	35+ 5	36+ 2	98.7- 99.3	9,400- 12200	7.1- 11.2	86- 102	120-150	2
7	33+ 4	34+2	98.4- 99.9	7,600- 14600	16.2- 13.9	86- 108	126- 170	5
8	34+ 4	35+ 1	98.5- 99.7	7,600- 13600	7.6- 9.9	80- 104	126-148	4
9	31+ 2	32+ 0	98.9- 100.2	6900- 15600	6.4- 10.3	87- 100	134- 157	5
10	34+ 5	35+ 2	98.4- 98.8	9,600- 12600	6.2- 10.7	78- 103	130- 170	4
11	35+ 3	36+ 0	98.7- 99.6	9,400- 12700	5.9- 10.9	76- 102	136- 170	4
12	30+ 4	30+ 5	98.9- 100.2	9,800- 15600	6.2- 10.5	78- 100	140- 150	4

**Figure 3:**
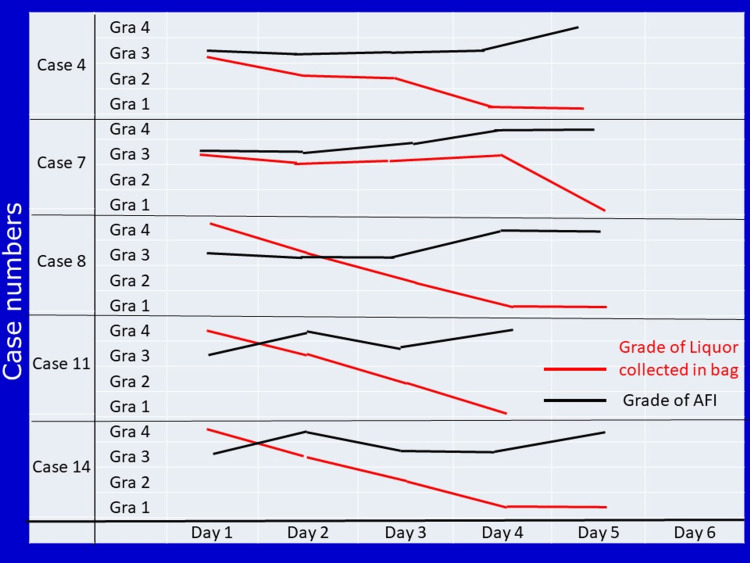
The trend of change in grade of collected liquor and grade of AFI in selected five women, in whom the leak could be stopped, is shown.

In 12 women, the leak did not stop. But we could continue pregnancies for a variable period of 2 to 5 days without a significant increase in parameters of infection (Table 2). We could utilize this period for the action of antenatal steroids. All these babies developed varying degrees of NRD and responded well to oxygen supplementation with masks for variable periods; and none of them required ventilator support. Birth weights ranged from 1.1 to 2.9kgs (Table 3). Five selected cases are shown in Figure 3.

**Table 3:** Perinatal outcomes of the 20 women who participated in the study. The clinical outcomes of women with PPROM who were treated with vaginal drainage are shown. AFOD: Amniotic fluid optical density; GA: Gestational age; NRD: Neonatal respiratory distress; NICU: Neonatal intensive care unit; TLC: Total leucocyte count, CRP: C-reactive protein.

Women, in whom the leak sealed								
SL. No.	AFOD on adm.	AFOD at Del	NRD	Mode of del.	NICU: Days	Birth weight	Preg. Cont. days	Perinatal outcome
1	0.18	0.56	Mild	LSCS	12	1.9	9	Good
2	0.11	0.43	Mild	ND	3	1.23	74	NND
3	0.35	1.51	Nil	ND	0	3.1	20	Good
4	0.45	0.66	Nil	LSCS	5	2.01	11	Good
5	0.11	1.23	Nil	ND	0	2.9	75	Good
6	0.21	0.67	Nil	LSCS	4	2.1	7	Good
7	0.31	0.62	Mild	LSCS	7	2.3	11	Good
8	0.21	0.49	Mild	LSCS	13	1.9	9	Good
Women, in whom the leak did not stop								
1	0.34	0.48	Mild	LSCS	12	1.9	3	Good
2	0.30	0.45	Mild	LSCS	11	1.8	3	Good
3	0.27	0.32	Mild	LSCS	3	2.4	4	Good
4	0.29	0.31	Mild	LSCS	3	2.5	3	Good
5	0.39	0.56	Mild	LSCS	35	1.1	4	NND
6	0.62	0.92	Nil	ND	0	2.9	2	Good
7	0.58	0.96	Nil	ND	10	2.1	5	Good
8	0.56	0.7	NIL	LSCS	5	2.1	4	Good
9	0.16	0.49	Mild	LSCS	13	1.9	5	Good
10	0.56	0.96	NIL	LSCS	6	2.2	4	Good
11	0.29	0.31	Mild	LSCS	3	2.5	4	Good
12	0.29	0.46	Mild	LSCS	22	1.4	4	Good

## Discussion

Preterm premature rupture of membranes (PPROM) is one of the most important causes of perinatal morbidity and mortality [13]. Prematurity predisposes to long-term morbidities like diabetes mellitus, hypertension, obesity, and stroke [5]. Ascending infection from the vagina results in dangerous chorioamnionitis, which threatens the life of the baby and mother. For the same reason, ‘Not to delay delivery for more than 24 hours after rupture of membrane’ is the thumb rule in obstetrics.

In this study, we used a vaginal drain, a novel method for preventing ascending infection. We could achieve complete cessation of leakage in 40% of women (8/20). We could continue pregnancies for a variable period of 7 to 74 days with these women. On the other hand, we could not stop leaks in 60% of women (12/20). But we could buy a period of 2 to 5 days without a significant increase in the parameters of infection. During this period, we could utilize antenatal steroids to act. 

By cleaning the vagina at the beginning of the procedure, we could reduce the bacterial load in leaked AF. Due to capillary action and also due to the gravity of the AF column in the drainage tube, the tip of Nelton’s urethral catheter sucks out the leaked AF immediately when it comes out. This prevents ascending infections and chorioamnionitis.

Effective tocolysis by low-dose Isoxsuprine Hydrochloride rapid infusion [10] and also Inj. Hydroxyprogesterone IM weekly stops/prevents uterine activity [10]. This further helps to reduce or stop pressure changes inside the uterine cavity. This helps to stop the movement of septic liquor material between the uterus and vagina. Complete bed rest with foot end elevation makes the fetus fall towards the chest and prevents cervical stimulation by the presenting part. All these mechanisms help to stop the leak and facilitate the spontaneous closure of the tear in the membrane.

We utilized the AFI measurement and the measurement of leaked AF collected in the bag to assess the status of the leaking membrane. We also utilized the AF samples collected from the drainage tube for AFOD estimation, which helped us decide on the time of delivery [11]. 

Four babies in the leak-sealed group and eight babies in the leak-not-sealed group developed mild NRD, which responded well to oxygen supplementation with a mask. None of them needed ventilator support (Table 3). 

This study is not without limitations. First, the sample size is small, and there is no control group for comparison. In this regard, an RCT involving a large sample size may be beneficial to prove the results. 

## Conclusions

By using a vaginal drain for P(P)ROM, ascending infection can be prevented, leaks can be stopped in 40% of women, and we can buy some time for ANS to act to prevent respiratory morbidity. As this is a small observational study, further multicentric randomized controlled studies are needed to confirm our results with a larger sample size. 
